# Impact of visual impairment on balance and visual processing functions in students with special educational needs

**DOI:** 10.1371/journal.pone.0249052

**Published:** 2022-04-29

**Authors:** Kai Yip Choi, Ho Yin Wong, Hoi Nga Cheung, Jung Kai Tseng, Ching Chung Chen, Chieh Lin Wu, Helen Eng, George C. Woo, Allen Ming Yan Cheong

**Affiliations:** 1 School of Optometry, The Hong Kong Polytechnic University, Hung Hom, Hong Kong; 2 Department of Optometry, Asia University, Taichung, Taiwan; 3 Research Centre for SHARP Vision (RCSV), The Hong Kong Polytechnic University, Hung Hom, Hong Kong; 4 Center for Eye and Vision Research (CEVR), Sha Tin, Hong Kong; University of Perugia: Universita degli Studi di Perugia, ITALY

## Abstract

**Introduction:**

Vision is critical for children’s development. However, prevalence of visual impairment (VI) is high in students with special educational needs (SEN). Other than VI, SEN students are prone to having functional deficits. Whether visual problems relate to these functional deficits is unclear. This study aimed to assess the impact of vision on visual processing functions and balance in SEN students through a community service.

**Methods:**

Visual acuity (VA) and contrast sensitivity were measured in a total of 104 (aged 14.3±4.3) SEN students as the visual outcomes, followed by retinoscopy. Visual processing function assessment included facial expression recognition by card matching examiner’s facial expression matching, and visual orientation recognition. Dynamic balance, by Timed Up and Go test, and static standing balance (postural sway in double-legged standing with feet-together and tandem-stance for open-eye and closed-eye conditions) were assessed. Static balance was presented in terms of the maximal medial-lateral and antero-posterior sways.

**Results:**

Of the 104 students, 62 (59.6%) were classified as visually impaired according to WHO classification of visual impairment based on presenting distance acuity. Ocular problems (e.g. optic nerve anomaly, uncorrected/ under-corrected refractive errors) and neurological anomalies were the major causes of vision loss. VA was positively associated with visual processing functions (all p ≤ 0.01), as SEN students with better vision tended to perform better in visual orientation and facial expression recognition tasks, as well as dynamic balance function (p = 0.04). For the static balance, postural sway and VA showed a positive relationship under open-eye and tandem stance conditions. However, the relationship between postural sway and VA became negative under closed-eye and tandem stance conditions.

**Conclusion:**

This study found a high prevalence of SEN students with visual impairment, in which many of them were undetected. Optometric examination is important to improve their visual function to minimize the effect of vision on functional performance. Vision is critical in visual processing as well as playing an important role in maintaining balance in SEN students.

## Introduction

Vision in children is crucial for their daily life, as it contributes greatly to the development of their functional abilities and essential skills needed for schooling and learning, such as reading comprehension, and mathematical concepts. However, visual disabilities are common in children with special education needs (SEN) [1]. Perinatal adversity is one of the major causes of vision loss [2,3], including preterm birth, improper neonatal environment, and neurological damage, which can affect visual acuity, contrast sensitivity, and ocular alignment. The level of visual impairment has been shown to be dependent on the severity of the SEN (e.g. grading of cerebral palsy) [4], which is partially attributable to cerebral visual impairment [5]. In addition to neurological and anatomical damage, there was also a high prevalence of correctable refractive error in children with SEN [6,7], so prescription of spectacles or other optical aids would be beneficial to improve their vision. However, recent evidence has indicated inadequate eye care services for children with SEN [8,9], leading to an inappropriate educational experience due to the misunderstanding of the individual’s visual status.

Balance function is also compromised in children with SEN, in terms of static and dynamic balance. Children with more severe cerebral palsy (higher gross motor function classification system level) recorded a greater magnitude of postural sway during static balance measurement than children with normal development [10], as well as dynamic balance function measured with the Timed Up and Go (TUG) test [11]. Children with Down syndrome tended to need more time to execute functional balance tasks, such as performing a standing reach [12] and the TUG test [13]. The postural control system was also found to be underdeveloped in children with autism spectrum disorder (ASD) [14] and deafness [15]. For children with other intellectual disabilities, it remains controversial whether their balance function is inferior to their peers with normal development [16,17]. However, visual impairment was associated with reduced balance function [17,18]. It was also reported that postural sway for male participants with visual impairment, regardless whether their eyes were open or closed, was similar to that of sighted participants with their eyes closed [19]. This implied that vision had less impact on postural control despite compromised balance function in visually impaired patients. Balance function in children with disruption of binocular vision, due to strabismus or amblyopia, was significantly reduced [20]. However, postural control was significantly improved after corrective surgery in children with strabismus [21], suggesting that there was a possibility to improve balance function by correcting vision or improved visual function. Children with visual impairment had poorer postural sway in both double-leg and single-leg standing compared with their sighted peers [22]. However, reduced static balance function was only found in eye-open, but not eye-closed condition. Both dynamic functional balance and coordination were also reported to be weaker in children who were visually impaired [23,24]. Despite the important role of vision on balance function, no studies have considered the impact of visual impairment in children who are more prone to have compromised balance function (e.g. children with SEN).

Visual processing is the ability of the brain to acquire, compute, and interpret visual information. In view of the importance of vision in childhood development, thorough assessment of visual function and related visual processing functions can provide information on the limitations of the SEN children [25]. Visuo-spatial and visuo-perceptual impairment, in terms of facial recognition and line orientation judgement, are common in children with bilateral cerebral palsy [26] and ASD [27,28]. Extremely pre-term birth or very low birth weight were associated with visual processing disorders and poorer academic performance in adolescents, after controlling for other perinatal risk factors [29]. However, based on the various capabilities of the SEN children, it has been suggested that evaluation for visual processing function could be better delivered in play situations [30], especially for children who functioned normally in other visual tasks, such as visual acuity [31]. Despite the importance of vision on learning different perceptual skills, it is unclear whether visual impairment further impairs visual processing performance of children with SEN.

Previous studies have suggested poorer balance function, weaker visual processing function, and higher prevalence of vision impairment in students with SEN. However, it is unclear whether compromised vision further impairs these SEN students’ balance and visual processing performance. The current study was based on the framework of a community outreach service project where assessments were conducted at the schools (a more familiar environment) for students with SEN instead of research laboratory or clinical settings. To our knowledge, this is the first study aiming to investigate the impact of vision on visual processing function and balance performance (dynamic and static) in students attending special-care schools in Taiwan. In spite of not being designed based on a hypothesis-driven research question, there were several implications retrieved from this project, which would be useful to share with other researchers, frontline teachers or clinicians.

## Methods

### Study population

This study analysed the cross-sectional data collected in Jun 2018. As part of a community service project to provide eye care services for beneficiaries of non-government organisations in Taiwan, students with special needs attending six special schools were recruited in the current study. Details of the schools are listed in S1 Table. Invitations were delivered to the students by the schools by convenience sampling. All tests were conducted by optometrists and trained university students. A total of 157 students agreed to participate in the study, of whom 127 had measurable visual acuity and at least one functional measurement. Students with solely visual impairment, but otherwise normal development (i.e., absence of other non-visual disabilities, n = 23) were excluded. The remaining 104 students (chronological age 14.3 ± 4.3 years, range 4–19 years, 43 females) were included in the analysis to assess the impact of vision on students with different disabilities, including cerebral palsy, ASD, Down syndrome, and other intellectual disabilities. Written informed consent and verbal assent (if feasible) were delivered by schools and obtained from the guardians and the students, respectively. All study procedures followed the tenets of the Declaration of Helsinki and were approved by The Hong Kong Polytechnic University Human Subjects Ethics Subcommittee.

### Data collection

Demographics and information on the disabilities were obtained by a structured questionnaire completed by either the guardians or school teachers. The questionnaire included birth history, types of disabilities, self-reported vision status, and visual problems. The subtypes of SEN were classified as cerebral palsy (CP), ASD, Down syndrome (DS), isolated intellectual disability (ID), and Others, which included deaf-mute, Rett syndrome, inborn errors of metabolism, etc.

Distance visual acuity was measured monocularly by Lea symbols at 3 m (or 1.5 m if vision was poor) using matching toys (The Good-Lite Company, USA). If the students were intellectually incapable of performing the test, tests with lower cognitive requirement were used sequentially: Cardiff acuity test at 1 m by pointing at the picture optotype; Cardiff acuity test at 1 m by preferential looking; and Lea gratings at 57 cm by preferential looking. Habitual visual acuity of the better-seeing eye was converted into LogMAR acuity recorded, and used for analysis, because the functional performances were assessed with visual correction aids. Refraction using retinoscopy was also performed. Near visual acuity was measured binocularly using a LEA near vision card (The Good-Lite Company, USA) in terms of LogMAR acuity. Binocular contrast sensitivity (CS) was measured using the letter version of Mars (The Mars Perceptrix Corp., US), or Lea low-contrast symbols flip-chart test / Hiding Heidi test (The Good-Lite Company, USA) at 40 cm if students were intellectually incapable, then LogCS was recorded. Types of visual acuity and CS measures are summarised in S2 Table. External and internal ocular health was assessed using slit lamp biomicroscopy and a direct ophthalmoscope (or binocular indirect ophthalmoscope with pupil dilation upon parent’s consent), respectively. Results of other visual functions, including visual fields, colour vision, binocular tests, and oculomotor tests, were included in the supplemental dataset.

Visual processing function was evaluated by two paediatric tests (The Good-Lite Company, USA). Visual orientation recognition was measured using the Lea mailbox game, in which the students were asked to drop a card through a slit opening in different orientations, and the average time for five trials was recorded (VO). Facial expression recognition was measured using the Heidi expression test. The test was divided into two parts: (1) expression recognition with the Heidi cards matching (FEC): students were given a set of expression cards, then they were asked to match the card with that the examiner displayed, (2) expression recognition with the examiner’s facial expression (FEE): students were given a set of expression cards, then they were asked to match the facial expression that the examiner expressed on his/her face. Both parts were timed, and the average time needed for the five trials was recorded.

Dynamic balance function was measured by the Timed Up and Go (TUG) test [32]. The students were asked to rise from a chair, walk three meters on a straight line, turn around, return to the chair, and sit down. The chair height depended on the height of the students using the common chairs provided by the schools, as the chronological age of the sample covered a wide range. The test was repeated three times, with the time needed for each trial being averaged and recorded. Static balance function, in terms of postural sway, was measured using a force plate (BP400600, AMTI, US) with double-leg feet-together standing and tandem stance. Double-legged feet-together standing was chosen to mimic the natural standing position in daily life, while tandem stance condition was chosen over one-leg standing to mimic the postural stability during adverse conditions because of the limited capability of the SEN students in the current study. Postural sway was measured at each condition while standing steadily for 20 s under each of the four conditions: (1) feet together + eyes open (FO); (2) tandem stance + eyes open (TO); (3) feet together + eyes closed (FC), and (4) tandem stance + eye closed (TC). Students were asked to fixate at a distant target at 3 m under eye open condition. The force plate measured the subjects’ position at the centre of pressure (COP), at which the maximum medial-lateral and antero-posterior sway (ML and AP–i.e. maximal amplitude of COP in the ML and AP dimensions) were generated and included in the analysis.

### Statistical analysis

As the distribution of some of the outcome variables was significantly different from normal, non-parametric tests were used in the analysis. Kruskal-Wallis test was used to compare visual functions, visual processing functions, and dynamic balance function among the subtypes of SEN (Table 1). Non-parametric Spearman’s partial correlation test, with chronological age as a covariate, was used to investigate the relationship of habitual visual acuity against VO, FEC, FEE (near), and TUG (distance), with Holm’s correction. For static balance, the results were transformed to achieve normality using percentile ranking followed by inverse-normal transformation into normally distributed Z-scores [33]. Multiple linear regressions were used to assess the relationship between visual acuity and sway in different eye and feet conditions, with the variance inflation factors (VIF) of the sway amplitude within 4 [34]. As the sample size was limited in the static balance measures, medial-lateral and antero-posterior sways were analysed separately with Holm’s correction. Inability to perform the test was regarded as missing data, which was treated with pairwise deletion. Static balance was not compared among subtypes of SEN because of the limited number of students who successfully completed different conditions in each group. Significance level was set as p < 0.05. All statistical procedures were performed using SPSS v22 (IBM Inc, US).

**Table 1:** Comparison of demographic, visual and functional performance among subtypes of SEN [median (IQR) or percentage].

	All	CP†	ASD†	DS†	ID†	Others‡	p-value
Demographic:	(n = 104)	(n = 18)	(n = 13)	(n = 12)	(n = 48)	(n = 13)	
Chronological age (in years)	16.0 (5.8)	16.0 (8.8)	13.0 (7.5)	17.5 (3.5)	17.0 (4.8)	**12.0 (3.5)**	**< 0.01**
Sex Female	43 (41.3%)	8 (44.4%)	3 (23.1%)	5 (41.7%)	20 (41.7%)	7 (53.8%)	0.61
Gestation age Full-term (≥ 37 weeks) Pre-term (< 37 weeks) Unknown	70 (67.3%) 17 (16.3%) 17 (16.3%)	9 (50.0%) **9 (50.0%)** 0 (0%)	12 (92.3%) 0 (0%) 1 (7.7%)	6 (50.0%) 2 (16.7%) 4 (33.3%)	33 (68.8%) 4 (8.3%) 11 (22.9%)	10 (76.9%) 2 (15.4%) 1 (7.7%)	**0.001**
Self-reported visual disabilities	31 (29.8%)	**11 (61.1%)**	2 (15.4%)	3 (25.0%)	13 (27.1%)	2 (15.4%)	**0.02**
Vision measures:							
Distance acuity of the better eye (LogMAR)	0.54 (0.55)	0.83 (0.73)	0.50 (0.73)	0.48 (0.38)	0.51 (0.66)	0.51 (0.25)	0.33
Near acuity (LogMAR)	0.40 (0.74)	0.40 (0.99)	0.27 (0.64)	0.45 (0.39)	0.46 (0.73)	**0.20 (0.19)**	**< 0.01**
Contrast sensitivity (LogCS)	1.62 (0.49)	1.64 (0.33)	1.90 (0.46)	1.60 (0.42)	1.60 (0.80)	1.60 (0.08)	0.39
Functional measures:							
Visual orientation–time (s)	1.78 (1.41)	1.58 (1.44)	1.94 (1.38)	1.90 (1.79)	1.91 (1.43)	**1.18 (0.62)**	**0.04**
Facial expression (Cards)–time (s)	11.88 (18.43)	20.77 (26.38)	13.33 (16.21)	11.75 (7.21)	11.00 (17.75)	**2.33 (3.63)**	**0.001**
Facial expression (Examiner)–time (s)	15.42 (29.32)	34.00 (48.19)	17.00 (100.33)	29.00 (37.25)	17.00 (26.17)	**4.60 (6.35)**	**0.001**
Time-up-go (TUG)–time (s)	11.72 (8.22)	15.36 (8.91)	10.60 (3.80)	17.69 (10.18)	13.22 (7.39)	**9.03 (1.91)**	**0.001**

† CP: Cerebral palsy; ASD: Autism spectrum disorder; DS: Down syndrome; ID: Intellectual disability.

‡ Others special educational needs include deaf-mute, Rett syndrome, inborn errors of metabolism, etc.

## Results

Of the 104 students, 62 (59.6%) fitted the WHO classification of visual impairment (VI) of having presenting-visual acuity ≥ 0.50 LogMAR in the better eye. The major causes of the presenting reduced vision (some students had multiple causes) were optic nerve related (N = 14, including optic atrophy, optic nerve hypoplasia, and glaucoma), retinal pathology (N = 7, including retinopathy of prematurity, retinal dystrophy, and macular anomalies), uncorrected/under-corrected refractive error (N = 11), ocular media opacity (N = 5), and oculomotor anomaly (N = 9). However, a large proportion of VI (N = 26) was probably not due to ocular problems, but other neurological causes (e.g., cerebral visual impairment, which refers to the vision loss caused by retro-geniculate damage in the absence of ocular abnormalities). Only 23 (22.1%) of the 104 students had optical correction before participating in this study. After subjective refraction or retinoscopy, 32 students were found to have uncorrected (24) / under-corrected (8) refractive errors and benefited from prescription of updated optical aids to improve their vision thereafter, with a mean improvement of visual acuity of LogMAR 0.33 ± 0.17. Notably, among the 62 presenting VI students, only 25 guardians/ teachers (40.3%) reported that they were aware of visual problems in their children/ students in the questionnaire, revealing an insufficient awareness of visual problems encountered in the SEN population. Given the different capabilities of the students, it was expected that some students could not complete all the tasks with a combination of reasons, including physical limitations, lack of understanding, visual problems, etc. The demographics, visual functions, visual processing functions, and balance performances in terms of the subtypes of SEN are listed in Table 1.

**Fig 1:**
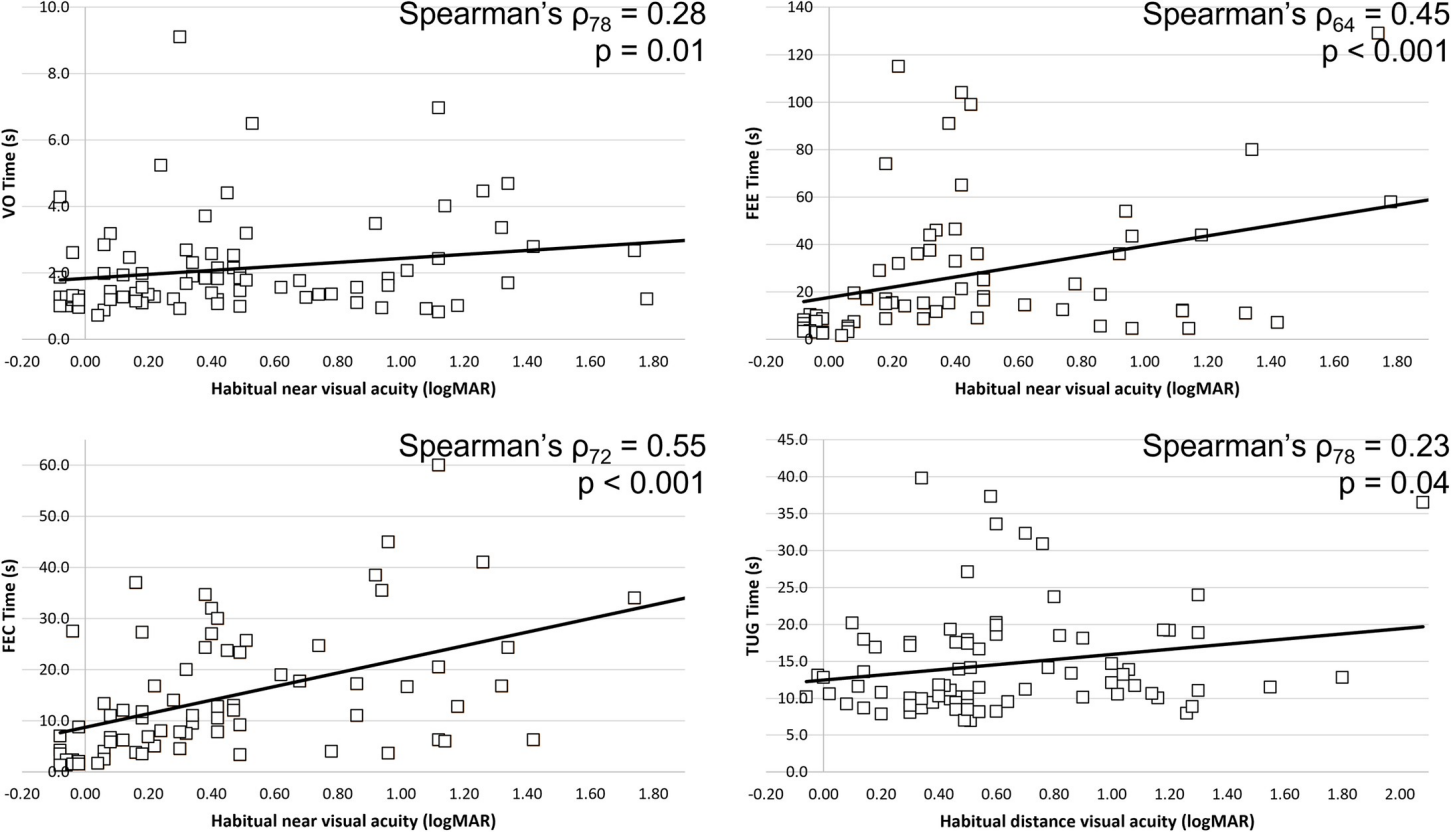
Relationship between visual acuity, visual processing functions (visual orientation (VO, N = 81), facial expression cards (FEC, N = 74), and facial expression examiner (FEE, N = 67), and dynamic balance function (time-up-go [TUG], N = 81). VO, FEC, and FEE were associated with habitual near acuity, while TUG was associated with habitual distance acuity.

In terms of vision, the chronological age was independent of distance visual acuity (Spearman’s ρ = 0.00, p = 0.98), near acuity (Spearman’s ρ = 0.21, p = 0.06), and contrast sensitivity (Spearman’s ρ = -0.18, p = 0.09). Chronological age was not significantly associated with performance in visual processing functions (Spearman’s test, p > 0.05). However, near visual acuity was significantly correlated with VO, FEC, and FEE, while distance visual acuity was significantly correlated with TUG after controlling for chronological age (Fig 1).

**Fig 2:**
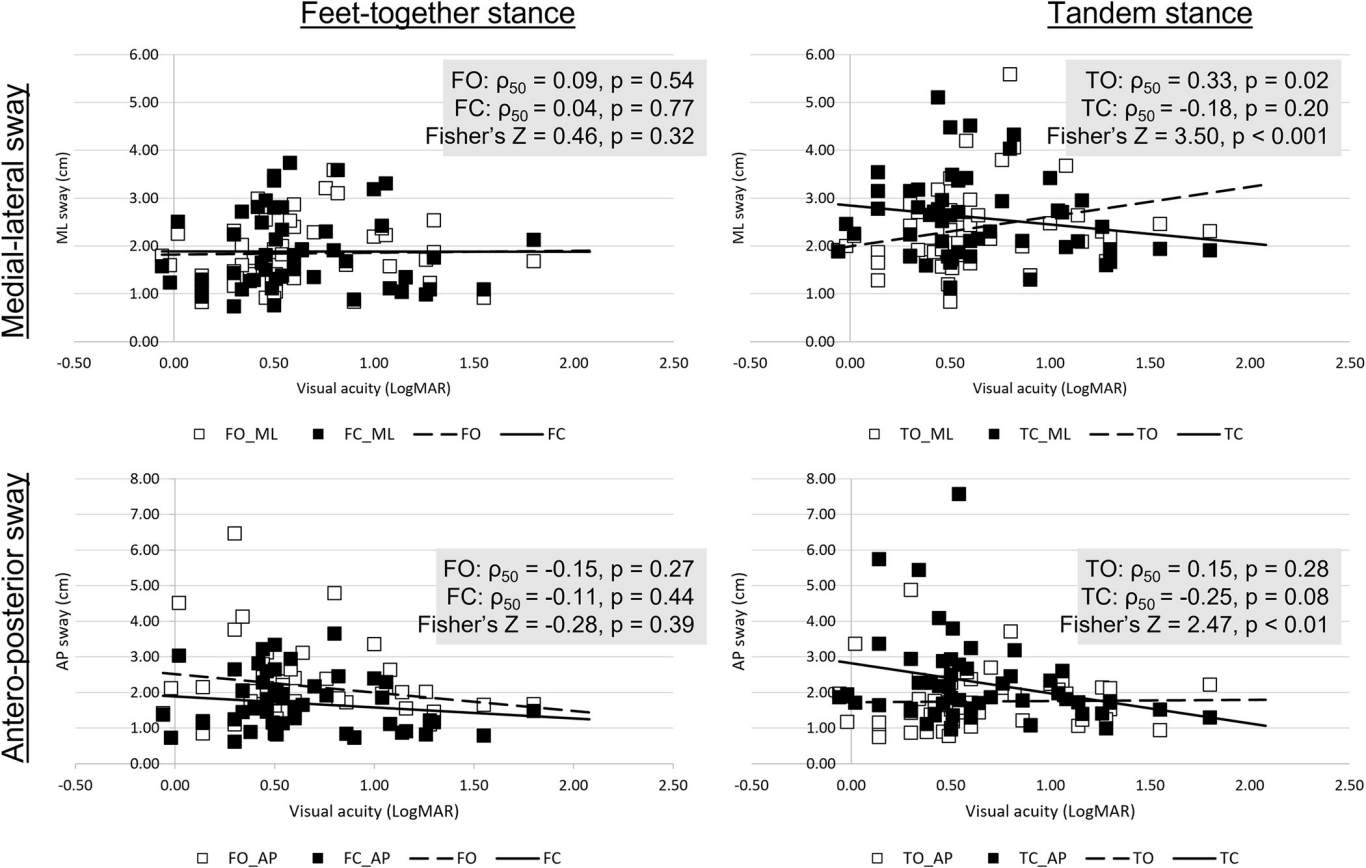
Relationship between distance visual acuity and static balance measures. Upper row: Medial-lateral sway (ML); Lower row: Antero-posterior sway (AP). Feet-together stance condition was denoted by F (left column, N = 52) while tandem stance condition was denoted by T (right column, N = 52). Open symbols indicate eye-open condition, while filled symbols indicate eye-closed condition. Statistical significance in Fisher’s r-to-z transformation indicates the different correlation coefficients.

In general, SEN students’ static balance function varied substantially when measured in different conditions. Results from the multiple linear regressions showed that the static medial-lateral and antero-posterior sways under feet-together conditions were independent from visual acuity in SEN students, regardless of whether their eyes were open or closed. In contrast, under tandem stance condition, the maximum sway amplitude increased with LogMAR acuity with eyes open, i.e., those who swayed more had poorer vision. In contrast, the maximum sway amplitude decreased with LogMAR acuity with eyes closed, i.e., those who swayed more had better vision (Fig 2). Detailed statistical results are listed in Table 2.

**Table 2:** Results of multiple linear regressions for static balance measures.

Condition	Unstandardised B	95% CI	Standardised B	p-value	VIF
	Medial-lateral sway (Adjusted R^2^ = 0.19, F = 3.95, p = 0.01)				
FO	-0.12	-0.28 to 0.04	-0.29	0.15	2.46
TO	0.28	0.12 to 0.43	**0.68**	**0.001**	2.36
FC	-0.01	-0.12 to 0.10	-0.03	0.84	1.47
TC	-0.15	-0.27 to -0.03	**-0.38**	**0.02**	1.51
	Antero-posterior sway (Adjusted R^2^ = 0.11, F = 2.55, p = 0.05)				
FO	-0.07	-0.21 to 0.08	-0.17	0.34	1.87
TO	0.15	0.01 to 0.30	**0.39**	**0.04**	1.88
FC	-0.09	-0.21 to 0.04	-0.25	0.16	1.78
TC	-0.09	-0.21 to 0.03	-0.23	0.14	1.30

F: Feet-together stance, T: Tandem stance, O: Eye open, C: Eye closed.

Statistics were based on normality-transformed values.

**Fig 3:**
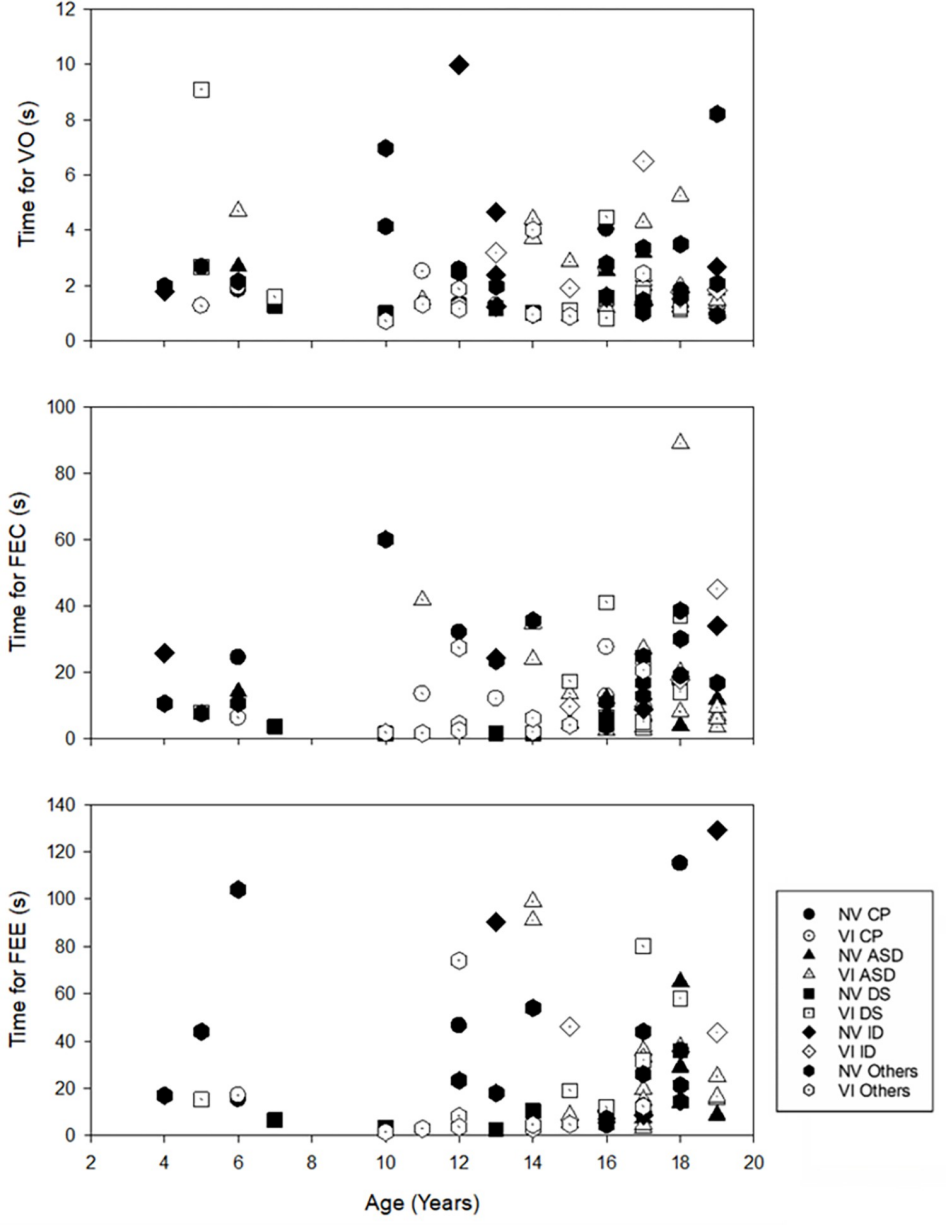
Relationship between visual processing functions and chronological age, stratified by visual acuity and subtypes of SEN. Filled symbols indicate students with visual acuity < LogMAR 0.50 (NV), while dotted symbols indicate students with visual acuity ≥ LogMAR 0.50 (VI). Circle: Cerebral palsy (CP); Triangle: Autism spectrum disorder (ASD); Square: Down syndrome (DS); Diamond: Isolated intellectual disability (ID); Hexagon: Other disabilities.

Given the wide spectrum of disabilities in SEN students among different subtypes of SEN, further analyses were conducted (Table 1, Fig 3). Chronological age differed significantly among the various subtypes of SEN (H = 16.21, p < 0.01). Students with SEN categorised as “Others” were significantly younger than those with DS (p = 0.02) and ID (p = 0.04), respectively by a median of 5.5 and 5.0 years. The habitual visual acuity and contrast sensitivity were similar among the subtypes of SEN (p > 0.05), but near visual acuity was better in Others (p < 0.01). However, students with different subtypes of SEN had significantly different performances in all visual processing function tests. For VO (H = 10.04, p = 0.04), students with Others performed significantly faster than those with ID (p = 0.03). These students also performed significantly faster than those with ID (p = 0.01), ASD (p = 0.01), and CP (p = 0.001) for FEC (H = 19.17, p = 0.001), and faster than those with ID (p = 0.01) and CP (p = 0.001) for FEE (H = 19.27, p = 0.001). Dynamic balance function also differed significantly among subtypes of SEN (H = 17.67, p = 0.001). Students with Others completed the TUG test faster than those with CP, DS, and ID (all p ≤ 0.01). Static balance function was not compared between SEN subtypes because of the limited number of students in each group. For example, only 2 of 13 ASD students could complete the static balance measurement due to difficulties in understanding or following the instructions.

## Discussion

In line with previous findings, we found a high prevalence of reduced visual acuity in students with SEN. Previous studies have reported an increased risk, as high as 75%, of visual impairment in disabled children [35], whereas the current study found that 59.6% of students attending the special schools met the WHO visual impairment criteria (LogMAR 0.50 or worse), although this figure did not include those SEN students with solely visual impairment (n = 23). In the US, cerebral visual impairment is the leading cause of visual impairment and blindness in children [36,37], and one study reported that this group constituted 19% of visually challenged children. Our study exceeded the findings of previous studies that the causes of reduced vision for 26/62 of the SEN students (41.9%) were likely to be neurological, while the remainder were due to different types of ocular anomalies (e.g. optic atrophy, glaucoma). Although the types of disabilities were self-reported by guardians or teachers rather than full medical records [38], it was speculated that cerebral visual impairment was likely to be the major cause of vision loss in students attending the special schools. Despite the high prevalence of visual impairment, the awareness of visual impairment of the guardians or teachers was still low, in that only 40.3% reported their children or students to have visual impairment. This implies the importance of optometric examination as uncorrected/ under-corrected refractive errors is one of the common causes of visual impairment in SEN students.

In current study, nearly one third of the SEN students were found to have uncorrected / under-corrected refractive errors, whose best-corrected visual acuity significantly could be improved by an average of LogMAR 0.33 by merely updating their spectacles. Although we did not measure accommodative functions, the high proportion of students requiring refractive correction (24 first-time prescribed, 8 updated, and 15 existing, totally 47 out of 104 [45.2%]) may be due to our East-Asian population having a higher prevalence of myopia [39]. This phenomenon indicates the necessity of regular eye examinations, as well as optical prescription, for the SEN population, as suggested in previous studies [8,9,40,41].

Vision loss has a significant impact on activities of daily living, affecting one’s functional performance and quality of life. Our findings showed that reduced visual acuity was significantly correlated with worse performances in functional tests, such as orientation recognition and facial recognition–two important visual processing tasks, despite the relatively low visual demand of these two functional tests. In addition, vision was significantly associated with balance function, both dynamic and static. SEN students with reduced visual acuity had poorer performance in the TUG test. Interestingly, it was also found that when the visual input was deprived (i.e. eye closed condition), visual acuity was negatively correlated with static balance sway amplitudes.

A significant relationship of visual processing functions with habitual visual acuity, despite the sizes of the cards used in both the Lea mailbox game and Heidi expression test were large (10.2 x 10.2 cm), requiring low visual demand to complete the tasks. This was evidenced with the relatively high completion rates for both tests (VO: 96/104; FEC 80/104). As in a previous study, visuo-spatial and visuo-perceptual abilities were found to be impaired in 90% and 60% of subjects with cerebral palsy, respectively [26], in terms of orientation judgement and facial recognition, which was independent of their visual acuity. Facial details processing deficits are also common in ASD [27,28], even if these individuals have normal vision, leading to problems such as unsustainable eye contact and switching focus for social function. Inhibition of visual input caused by visual impairment might have limited impact on such social information. In the current study, better performance in VO, FEC, and FEE were associated with better habitual visual acuity. However, chronological age did not show a significant association with the performance in visual processing functions. In line with previous studies, our results indicated that non-visual disabilities, e.g., intellectual disability, might account for the impaired visual processing functions rather than actual vision. To facilitate the learning of the SEN students with visual impairment, training tools of sufficient size should be employed to accommodate their visual needs. Further studies on functional assessments, such as joint attention and imitation, are warranted. In addition, the current study focused on orientation judgement and facial expression recognition. However, other types of visual processing functions, e.g., visual discrimination, visual memory, spatial relations, visual-motor, and visual-auditory integration, are also critical for development in SEN students. Further exploration of the relationship between subtypes of SEN, visual functions, optical corrections, and visual processing functions would facilitate the design of training/learning modules and benefit the development of the SEN students.

The balance functions of SEN students are reduced, regardless of the type of disability. In children with normal development, the dynamic balance function measured by TUG ranged from approximately 4 to 7 s [13,42]. The time needed to complete the TUG task increased from a median of 7.5 s in gross motor function classification system level I, to 17.8 s in level II, and finally 50.7 s in level III for children with CP [11], while it was approximately 9 s in adolescents with DS [13]. Our sample recorded a median of 11.72 s in SEN students (IQR 8.22 s). The correlation analysis showed a significant, but weak association between habitual visual acuity and TUG (Spearman’s ρ = 0.23, p = 0.04). This indicated that visual and non-visual disabilities compromised SEN students’ dynamic balance function in different ways and might have a composite effect, as dynamic balance function was further reduced in students with both visual and non-visual disabilities. Several studies have compared static balance function in sighted and visually impaired participants. In summary, they concluded that visually impaired individuals had greater postural sway than sighted individuals under open eye condition, especially in single-leg-standing positions: adults [19,43], adolescent [17], and children [18,22]. However, no significant difference was observed between the sighted and visually impaired individuals under closed eye condition. In the current study, a similar outcome was found in the SEN students. Those students with better visual acuity had better balance performance in open eye condition than those with poorer visual acuity under tandem stance, while no significant difference was observed in feet together standing. When visual input was deprived (i.e., eye closed condition), their static balance function became significantly poorer. However, students with both visual and non-visual disabilities swayed similarly under eye open and eye closed conditions. It is speculated that students with visual impairment may rely more on their somatosensory and/or vestibular inputs rather than vision to maintain their postural control, while students with better vision rely more on visual input. Coincidently, it was observed that similar balance performance was found in older adults with visual impairment [44]. They swayed significantly more than subjects with normal vision in static standing under eye open, but displayed no difference with sighted aged-matched subjects when eyes were closed. The reliance on somatosensory input was shown when subjects with visual impairment stood under a sway-reference support surface with eye open swayed significantly more than the sighted subjects [44]. Further studies examining the contribution of multi-sensory inputs to postural control, and functional activities, such as obstacle avoidance and crossing, are needed. Such knowledge could facilitate the design of balance training protocols for students with SEN.

Taiwan initiated a national-wide registry policy for children with disabilities in the 1980’s to better understand and provide support for those with SEN. The combined prevalence of SEN has increased from 1.0% to 1.5% over three decades [45], affecting more males, especially in rural areas [46]. Institutionalised care, e.g. special schools, has been the mainstream for the special care services [47], providing educational, occupational, and vocational training for registrants. Although resources had been pooled for rehabilitation services, the prevalence of the beneficiaries was still low [48], in which the service recipients only accounted for 24.5% of an 957-subject SEN sample within a 7-month period. As well as the students with SEN themselves, the primary family carer may also experience challenges, causing the carers’ health status and quality of life to be significantly lower [49]. In the current study, despite the students attending special care schools, awareness of visual impairment of the guardians or teachers was still low, in that only 40.3% reported their children or students to have visual impairment. The well-established SEN care in Taiwan allows parents to have easy access to clinical assessments and support. However, in an unfamiliar environment, students with SEN may not reflect their usual performance of tasks to assess their abilities. In such cases, outreach service may be a better alternative, in which the SEN student would be calmer than being in a hospital or clinical setting. Also, entertaining game-like tests, such as the ones in the current study, can be applied to attract their attention rather than conventional tests.

There were several limitations in the current study. Firstly, the self-reporting disability status was less reliable than reviewing a full medical history. However, due to the compliance with patient privacy, this information could not be retrieved from the guardians or teachers. Secondly, the severity of non-visual disabilities was not assessed in the current study, which is speculated to affect the capability of SEN student to perform different tasks. Most of our tests required students to have a certain degree of cognitive ability to participate. It is not surprising that almost half of the participating students could not complete the various conditions in balance measurement, in particular the tandem stance in eye closed condition. While most previous studies categorised their subjects into groups to indicate their visual status, the current study directly investigated the relationship between visual acuity, visual processing functions, and balance functions. However, our results on visual processing functions achieved statistical power of 0.89, 1.00, and 1.00 for VO, FEC, and FEE, respectively. For the balance functions, our results achieved 0.76, 0.96, and 0.80 statistical power for TUG, ML sway, and AP sway, respectively. The relationships between visual acuity and visual processing functions were reported as univariate correlations owing to our limited scale of study, which might be confounded by other factors, including other visual functions and systemic effects of cerebral visual impairment and cerebral palsy. Further studies are needed to perform a more comprehensive multivariate analysis to obtain the whole picture. Finally, this study lacked a longitudinal follow-up to observe the effect of improved vision after updated optical corrections on visual processing functions (both those measured in the current study and others, including reading speed, visual memory, and spatial relations) and balance functions in SEN students.

To conclude, visual impairment in SEN students is common. However, despite the high prevalence of visual impairment in this population, our findings suggest that some SEN students’ visual function could be improved by prescribing appropriate and updated refractive correction. Vision is an important integral for children’s all-round development, including that of SEN students. Parents and teachers could use tools of sufficient size for training and learning purposes to help overcome their visual impairment. Adequate visual input was also found to be critical for SEN students to maintain their dynamic and static balance functions. Hence, regular checking and preserving of the vision of SEN students is of high importance.

## Supporting information

S1 Table Participating schools. (DOCX)

S2 Table Breakdown of sub-types of visual acuity and contrast sensitivity measurements. (DOCX)
