# Correction: Impact of visual impairment on balance and visual processing functions in students with special educational needs

**DOI:** 10.1371/journal.pone.0293152

**Published:** 2023-10-12

**Authors:** Kai Yip Choi, Ho Yin Wong, Hoi Nga Cheung, Jung Kai Tseng, Ching Chung Chen, Chieh Lin Wu, Helen Eng, George C. Woo, Allen Ming Yan Cheong

There are errors in the Funding section. The correct Funding statement is: This study was supported by four different funds: 1) Community Service Fund (83CT), The Hong Kong Polytechnic University; 2) Chinese Mainland Affairs Office Fund, The Hong Kong Polytechnic University; 3) Lions Clubs International MD300 Taiwan Eye Care Network Committee; and 4) InnoHK, HKSAR Government.
