# Supplementary material for: Impact of visual impairment on balance and visual processing functions in students with special educational needs
Source: PLoS One. 2022 Apr 29;17(4):e0249052. doi: 10.1371/journal.pone.0249052 (PMC9053808; doi:10.1371/journal.pone.0249052)
Supplement: S1 Table — (DOCX) [file pone.0249052.s001.docx]

S1 Table. Participating schools

| Name of schools |
| --- |
| 臺中市立啟明學校  (Taichung School for the Visually Impaired, <http://www.cmsb.tc.edu.tw/home>) |
| 高雄市立仁武特殊教育學校  (Kaohsiung Municipal Renwu Special Education School, <http://school.kh.edu.tw/view/index.php?WebID=253&MainType=HOME>) |
| 高雄市立成功啟智學校, currently known as高雄市立成功特殊教育學校  (Kaohsiung Municipal Chenggong Special Education School, <http://school.kh.edu.tw/view/index.php?WebID=283&MainType=HOME>) |
| 國立彰化特殊教育學校  (National Changhua Special School, <https://www.chsmr.chc.edu.tw/bin/home.php>) |
| 私立惠明盲校  (Huei-Ming School, <http://www.hmsh.tc.edu.tw/>) |
| 惠明盲童育幼院  (Huei-Ming Nursery, <https://hueiming.eoffering.org.tw/>) |
