# Supplementary material for: Impact of visual impairment on balance and visual processing functions in students with special educational needs
Source: PLoS One. 2022 Apr 29;17(4):e0249052. doi: 10.1371/journal.pone.0249052 (PMC9053808; doi:10.1371/journal.pone.0249052)
Supplement: S2 Table — (DOCX) [file pone.0249052.s002.docx]

| **Distance visual acuity** | *(in LogMAR)* |  |  |
| --- | --- | --- | --- |
|  | Lea symbols | Cardiff acuity test | Lea gratings ^§ ¶^ |
| N | 67 | 32 | 5 |
| First quartile | 0.34 | 0.30 | 0.97 |
| Median | 0.50 | 0.60 | 1.36 |
| Third quartile | 0.86 | 0.90 | 1.78 |
| § and ¶ indicates the significant difference from Lea symbols and Cardiff acuity test, respectively | | | |
|  |  |  |  |
| **Contrast sensitivity** | *(in LogCS)* |  |  |
|  | MARS^§ ¶^ | Lea low contrast symbols flipchart | Hiding Heidi test |
| N | 54 | 19 | 23 |
| First quartile | 1.29 | 1.00 | 1.90 |
| Median | 1.60 | 1.90 | 1.90 |
| Third quartile | 1.64 | 1.90 | 1.90 |
| § and ¶ indicates the significant difference from Lea low-contrast symbols flipchart and Hiding Heidi test, respectively | | | |

S2 Table. Breakdown of sub-types of visual acuity and contrast sensitivity measurements
